# Editorial: Glial crosstalk in neurological disorders

**DOI:** 10.3389/fcell.2024.1515052

**Published:** 2024-11-12

**Authors:** Ikuko Miyazaki, Masato Asanuma, Francisco Javier Díaz-Corrales

**Affiliations:** ^1^ Department of Medical Neurobiology, Graduate School of Medicine, Dentistry and Pharmaceutical Sciences, Okayama University, Okayama, Japan; ^2^ Retinal Neurodegeneration and Advanced Therapies, Integrative Pathophysiology and Therapies Department, Andalusian Molecular Biology and Regenerative Medicine Centre (CABIMER), Seville, Spain

**Keywords:** glia, astrocyte, microglia, oligodendrocyte, neurological disorder, glial interaction

Neurons have been focused as a main target for investigation of pathogenesis and therapeutics in neurological disorders. Although some of the mechanisms, including oxidative stress and inflammation, followed by apoptosis, are thought to be involved in the pathogenesis of these diseases, pathological mechanism remains unknown. There is a consensus on the involvement of non-neuronal cells in the pathological progression. Recently, glial cells are getting attention as a key players of non-cell autonomous neurodegeneration in neurological disorders. Especially, glial crosstalk and its action on neurons are highlighted. It is demonstrated that microglia convert astrocytes to neurotoxic reactive A1 astrocytes (Liddelow, et al., 2017). In contrast, it is also reported that astrocytes activate microglia to produce neuroinflammation (Rohl and Sievers, 2005). Besides, astrocyte-microglia crosstalk contributes to degradation of protein aggregates (Rostami, et al., 2021). Furthermore, recent studies indicated microglia-oligodendrocyte or astrocyte-oligodendrocyte interaction promoted neuronal dysfunction and neurodegeneration (Lohrberg, et al., 2020; Papazian, et al., 2021). Thus, glial communication could be a main target to understand pathological mechanism and develop neuroprotective therapeutic approach. This series includes five articles covering different aspect of the Research Topic outlined above.

The review by Shigetomi et al. focuses on the mechanisms used by neurons and glia to cooperatively produce the activity-dependent increase in ATP/adenosine and its physiological and pathophysiological significance in the brain. Neuronal activity and brain insults such as ischemic and traumatic injury upregulate extracellular ATP/adenosine, which exerts their effects by activating purinergic receptors. The authors described methods for analyzing extracellular ATP/adenosine dynamics as well as the current state of knowledge on the spatiotemporal dynamics of ATP/adenosine in the brain.

The mini review by Xu et al. summarizes the role of microglia during brain development and describes microglial roles after viral infection through microglia-neural crosstalk. Environmental factors, such as infection and stress alter microglial phenotype and function. Viral infection activates microglia to produce inflammatory cytokines and anti-viral responses of microglia protect brain from damage. The authors also discuss limitations for current studies and highlight future investigated questions.

The paper by Jeon et al. reports visualization of perivascular macrophages and microglia in the retinal ganglion cell layers using cx3cr1-GFP (C57BL6) transgenic mice with both healthy and disease conditions including NaIO_3_-induced retinal degeneration models and inter-photoreceptor retinoid-binding protein-induced auto-immune uveitis models. The authors found two subsets’ microglia in the ganglion cell layer; peripheral microglia located on the retinal parenchyma and BAM (CNS border associated macrophage) which have a special stretched phenotype only located on the surface of large retinal veins.

The paper by Lu and Hyde reports the essential role of inflammatory cytokines IL-1β and IL-10 on Müller glia proliferation following light damage in adult zebrafish. Resident Müller glia respond to damage by reprogramming and undergoing an asymmetric cell division to generate a neuronal progenitor cell. In contrast, microglia become reactive, phagocytose dying cells, and release inflammatory signals into the surrounding tissue following damage. The authors demonstrated inflammatory cytokines expression during retinal regeneration after light damage.

The review by Akinlaja and Nishiyama focuses on the glial modulation of synapse development and plasticity. Glial cells have been identified as crucial participants in influencing neuronal activity and synaptic transmission, with astrocytes forming tripartite synapses and microglia pruning synapses. The authors describe the roles of different glial cell types at synapses, including the recently discovered oligodendrocyte precursor cells and glial cross-talk in pathological states such as schizophrenia, dementia disorders and glioma.

We hope that this Research Topic can produce discussion about therapeutic strategies that glial crosstalk as a target for the development of disease-modifying therapies for neurological disorders in the future. Finally, we would like to take this opportunity to express my gratitude to the all authors and co-authors for their excellent contributions to this Research Topic.
